# Harnessing gastrointestinal microbial co-association networks to predict feed efficiency and methane emissions across beef and dairy cattle

**DOI:** 10.1099/mgen.0.001689

**Published:** 2026-04-13

**Authors:** Pamela A. Alexandre, Yuliaxis Ramayo-Caldas, Milka Popova, Ioanna-Theoni Vourlaki, Gilles Renand, Aurélie Vinet, Diego P. Morgavi, Antonio Reverter

**Affiliations:** 1 CSIRO Agriculture and Food, St. Lucia, Brisbane, Queensland 4067, Australia; 2 Animal Breeding and Genetics Program, IRTA, Torre Marimón, 08140 Caldes de Montbui, Barcelona, Spain; 3 Université Clermont Auvergne, INRAE, VetAgro Sup, UMR Herbivores, Saint-Genes-Champanelle, France; 4 Université Paris Saclay, INRAE, AgroParisTech, GABI, 78350 Jouy-en-Josas, France

**Keywords:** amplicon sequencing, Charolais, Holstein, rumen liquid and faecal microbiota

## Abstract

Enteric methane emissions from cattle pose a significant environmental concern and represent a substantial energy loss for the animal, necessitating the development of effective mitigation strategies. The gastrointestinal microbiota plays a crucial role in determining both feed efficiency and methane production. Still, the specific microbial signatures that predict these traits across different production systems remain poorly understood. This study aimed to identify common predictive microbial biomarkers for feed efficiency and methane emissions using co-association network analysis across contrasting cattle production systems. Rumen liquid and faecal microbiota from 55 Charolais heifers (beef) and 56 Holstein cows (dairy) were analysed using 16S rRNA gene amplicon sequencing. Phenotypic data included feed efficiency, methane yield and acetate/propionate ratio. Co-association networks were constructed using Partial Correlation and Information Theory to identify amplicon sequence variants (ASVs) directly connected to phenotypes. Multiple regression analysis determined the minimal ASV sets required to achieve optimal predictive accuracy. Rumen microbiomes consistently showed superior predictive performance compared to faecal communities across all traits. Network-selected ASVs explained substantial phenotypic variance across traits and production systems (*R*²=0.45−0.84), consistently outperforming randomly selected ASVs by 0.13–0.44 *R*² units. The ACET:PROP ratio showed the highest predictive accuracy (*R*²=0.84 in Charolais rumen, 0.77 in Holstein rumen), while minimal ASV sets achieved 93–97% of the full model's performance using 33–65% fewer ASVs (6–17 ASVs). *Bacteroidaceae* was consistently enriched across phenotypes in rumen networks, regardless of production system. Contrary to expectations, most associations were production system-specific, with notable exceptions including negative correlations between the ACET:PROP ratio and *Prevotella*/*Ruminococcus* genera and negative associations with members of the *Succinivibrionaceae* family for methane-related traits. The anatomical site-specific and production system-specific nature of most associations underscores the importance of context-specific approaches.

**Impact Statement**

This research advances precision livestock management by demonstrating that network-selected microbial signatures have the potential to predict feed efficiency and methane emissions in cattle. The study identifies specific taxa, such as *Succinivibrionaceae* family members, that show consistent negative associations with methane-related traits across production systems, providing potential biomarkers for developing environmental mitigation strategies and early screening tools in breeding programmes. However, the predominantly production system-specific nature of most associations indicates that universal microbial biomarkers may be limited, requiring context-specific calibration for different breeds and management systems rather than a universal approach.

## Data Summary

Fifty-five Charolais beef heifers and 56 Holstein dairy cows. Dual sampling sites (rumen liquid and faecal microbiota). 16S rRNA gene amplicon sequencing (V3–V5 region) resulting in 6,867,844 quality-filtered reads and 6,032 amplicon sequence variants identified. Three phenotypes: feed/milk efficiency (production output per dry matter intake), methane yield (methane production per dry matter intake) and acetate/propionate ratio (proxy for ruminal fermentation patterns). Supplementary material is available on Figshare at DOI: 10.6084/m9.figshare.31442305 [1]

The data that support the findings of this study are available from the European Nucleotide Archive under the project PRJEB98694 (https://www.ebi.ac.uk/ena/browser/view/PRJEB98694). Accession ID and details for individual files are available in File S1.

## Introduction

The beef and dairy industries play a pivotal role in global food security and the economy [2]. However, the environmental impact of livestock production, particularly the release of methane (CH4), has drawn increasing attention due to its role as a greenhouse gas, impacting consumers’ trust and farmers’ social licence to operate [3]. Efforts to reduce methane emissions and improve production efficiency in ruminant livestock require a comprehensive understanding of biological processes that control these phenotypes. This includes the microbial communities residing in the gastrointestinal tract, as they are responsible for methane production and significantly influence nutrient utilization.

Early microbiome-wide association studies in pigs identified several operational taxonomic units (OTUs) that were significantly associated with performance and health traits [4,5]. By analogy to the polygenic determinism of traits, it was suggested that these traits could also have a polymicrobial nature. Importantly, it is expected that the contribution of host genetics vs. microbiome profile varies depending on the specific trait being examined, the anatomical localization of the microbiome and the ecological context [6]. For example, research by Camarinha-Silva *et al*. on the gastrointestinal microbiota revealed that traits such as feed conversion ratio and feed intake in pigs show higher estimates of microbiability compared to their corresponding heritabilities. Conversely, in dairy cattle, Difford *et al.* [7] found higher heritabilities for methane emissions compared to microbiability when examining rumen microbial composition, despite methane having a microbial origin. These findings underscore the importance of recognizing the variability in gastrointestinal microbiome composition and its association with performance traits. It also opens the exciting possibility of predicting phenotypes based on microbial information at an earlier stage in an animal’s productive life. However, it is essential to note that not all microbes are heritable nor contribute to host traits [6].

Despite a growing body of research on the role of the microbiome in livestock production, inconsistencies and variations have emerged across microbiome studies. These discrepancies may arise from a combination of technical and biological factors, including differences between studies in sequencing technologies, taxonomic classification databases and the limited availability of FAIR (Findable, Accessible, Interoperable and Reusable) and comparable public datasets [8]. Additionally, the intricate interactions among host genetics, diet and environmental conditions further complicate efforts to identify the key microbial taxa that influence phenotypic traits such as methane emissions and production efficiency. Recently, Zhao *et al.* [9] demonstrated that integrating metagenomics, metatranscriptomics and metabolomics provides superior insights into active microbial processes. Importantly, network-based approaches have emerged as powerful tools for identifying keystone micro-organisms and functional modules within complex microbial communities, moving beyond simple correlation analyses to identify genuine biological dependencies rather than isolated entities [9].

In this study, we employed network analysis to investigate the potential of cattle gastrointestinal microbiota in predicting the efficiency of converting ingested feed into either milk or meat, as well as the associated methane yield. By concomitantly analysing data from two different production systems, we also hypothesized that common micro-organisms capable of predicting these traits could be identified. The identification of key predictive micro-organisms can facilitate the testing of animals through targeted approaches and the incorporation of microbiome information into predictive models in animal breeding.

## Methods

### Datasets

Two datasets were used for this study, one comprising 55 Charolais (beef) heifers (CHA) and the other comprising 56 Holstein (dairy) cows (HOL). Both datasets contained 16S rRNA gene amplicon sequencing data from rumen liquid and faeces, as well as phenotypic data for feed efficiency or milk efficiency, methane yield (CH4Y) and rumen acetate/propionate ratio (ACET:PROP) (Table 1), as described below.

**Table 1.** Summary statistics for feed efficiency (EFF), methane yield (CH4Y) and acetate/propionate ratio (ACET:PROP) for Charolais heifers and Holstein cows

		EFF	CH4Y	ACET:PROP
Charolais heifers	*N*	55	55	55
	Mean	0.97	24.95	4.92
	Std	0.22	4.11	0.42
	Min	0.54	17.33	3.96
	Max	1.58	37.45	5.79
Holstein cows	*N*	56	56	56
	Mean	1.46	22.99	4.17
	Std	0.15	2.20	0.40
	Min	1.14	18.82	3.14
	Max	1.77	28.11	4.88

CHA EFF=average daily gain (kg) divided by average dry matter intake (kg).

HOL EFF=average milk production (kg) divided by average dry matter intake (kg).

CH4Y=methane production (g) divided by average dry matter intake (kg).

CHA animals are a subset of the samples employed by [10] for which feed efficiency was calculated as average daily gain (kg) divided by average dry matter intake (DMI) (kg) during the evaluation period [10]. Milk efficiency in HOL was calculated as milk production (kg) divided by DMI [11]. Methane production (CH4) was measured using two GreenFeed Emission Monitoring (GEM) systems (C-Lock Inc.). CH4Y for both datasets was calculated as CH4 divided by DMI. Concentrations of individual volatile fatty acids (VFAs) were analysed by gas chromatography with a flame ionization detector [12]. Notably, although both datasets contain similar phenotypes and microbiota data, each represents a vastly different production system. Charolais heifers were fed a roughage diet during the test period, and Holstein cows were fed a total mixed ration, consisting of maize silage and soybean cake. Please refer to [10] and [11] for more information about animal management and data collection.

### Amplicon data processing

For both datasets, amplicons were generated using the same method, with primers targeting the V3–V5 region of the bacterial 16S rRNA gene (F357 and R926), and sequenced on a MiSeq sequencer for 251 paired-end cycles. Although the two datasets were sequenced in different runs, the generated reads were processed with the same pipeline using QIIME 2 (Quantitative Insights Into Microbial Ecology) version 2022.8 [13]. Because of insufficient overlap between forward and reverse reads in some samples, we analysed single-end data. Raw sequence data were quality filtered using q2-cutadapt for adapter removal and q2-dada2 for denoising [14]. Singletons were removed, and amplicon sequence variants (ASVs) were retained for downstream analyses. Taxonomy was assigned using a naïve Bayes classifier trained on the Greengenes2 reference database [15], after extracting the amplicon region corresponding to the primers used. Alpha and beta diversity metrics were computed using the online tool MicrobiomeAnalyst 2.0 [16] using default filtering parameters. An ASV was considered part of the core microbiome if it was present in at least 50% of the samples within each combination of breed × sample site.

The ASV counts were normalized using cumulative sum scaling (CSS) with the MetagenomeSeq [17] R package and then log2-transformed after adding a pseudo count of 1. Afterwards, Link-HD [18] was employed to compare the transformed input data with that of a centred log-ratio (CLR) transformed data using the vector correlation coefficient (RV) (Escoufier, 1973). A remarkably high and strong RV of 0.999 was observed between the two. Thus, indicating that the geometry of our CSS-log2 transformed data closely aligns with the CLR compositional transformation.

Because both datasets contained animals sampled in two different weeks, a permutational multivariate ANOVA was used to assess this effect on the ASV abundances. To do that, we used adonis2 from the R package Vegan using the Bray–Curtis method and 999 permutations [19]. No significant effect of sampling week was observed (*P*>0.05).

### ASV selection

To build co-association networks, we divided the dataset into Charolais rumen (CHA_Ru), Charolais faeces (CHA_Fe), Holstein rumen (HOL_Ru) and Holstein faeces (HOL_Fe). Each of the four networks contained phenotypes for each animal as well as ASVs with abundance values for at least 20 samples (Files S2–S5, available in the online Supplementary Material). This prevalence filter reduces sparsity and ensures that associations are estimated from enough non-zero observations. The networks were created using the Partial Correlation and Information Theory algorithm [20], which evaluates all possible three-way combinations of features. For each trio, PCIT computes the three first-order partial correlations and derives a tolerance value that represents the expected magnitude of indirect associations. An observed correlation is retained only if it exceeds this tolerance and, therefore, cannot be explained by the third variable in the trio. This procedure identifies direct, conditionally independent interactions. ASVs directly connected to each phenotype (ASV–phenotype connections) were considered potential predictors of the phenotype.

To explore the functionality of microbes associated with the phenotypes, we used the OTU2Taxa function from the LinkHD framework [18]. This function aggregates OTUs into their taxonomic characteristics and returns the most significant family, in this case, from a list of selected ASVs compared to a background. For each family, the function returns the hypergeometric distribution function *P*(*x*≥*X*) for each count and indicates those with counts greater than one. The higher the returned value, the higher the enrichment of that family. We assessed the ASVs significantly associated with each phenotype in each network against a background defined by all the ASVs included in that network.

### Predictive ability

To evaluate the ability of these ASVs to predict the phenotypes [feed efficiency (EFF), CH4Y and ACET:PROP], we performed a multiple regression analysis using the PROC REG programme (SAS Inst. Inc.) and choosing the RSQUARE method (selection=rsquare, best=1). This method performs regression on all possible subsets by incrementing the number of independent variables by one until all combinations are tested. It then displays the single best model for each number of predictor variables, in increasing order of *R*-squared magnitude. This way, ASVs that explain the most phenotypic variance are added sequentially. Because functional redundancy is a common microbial feature, we compared the *R*-squared value of the full model (which includes all ASVs) to that of every other subset model tested to determine the number of ASVs required to explain ~95% of the phenotypic variance explained by the full model. In addition, considering the number of ASVs associated with each phenotype, we randomly sampled the same number of ASVs and evaluated them as predictors of the respective phenotype. We repeated this process ten times for each trait × dataset combination. We did this to test the ability of the co-abundance networks to capture relevant ASVs relative to a random selection. Finally, we implemented a 5-fold cross-validation (CV) scheme for the selected ASVs, in which, for each fold, a regression model was fit on ~80% of the data (~44 animals, training population) and used to predict the held-out ~20% (~11 animals, validation population). For each fold, we computed CV-*R*² as the square of the Pearson correlation between observed phenotypes and predictions in the validation population.

## Results

### Microbial community composition and diversity patterns

The sequencing of all 222 samples across two production systems and 2 body sites (rumen and faeces) yielded a total of 6,867,844 reads after primer sequence removal and quality filtering. Denoising and clustering of non-chimeric reads resulted in the identification of 6,032 ASVs across all samples, ranging in length from 233 to 250 bp and excluding mitochondrial reads and singletons. The complete taxonomic classification of individual ASVs is provided in File S6.

**Fig. 1.**
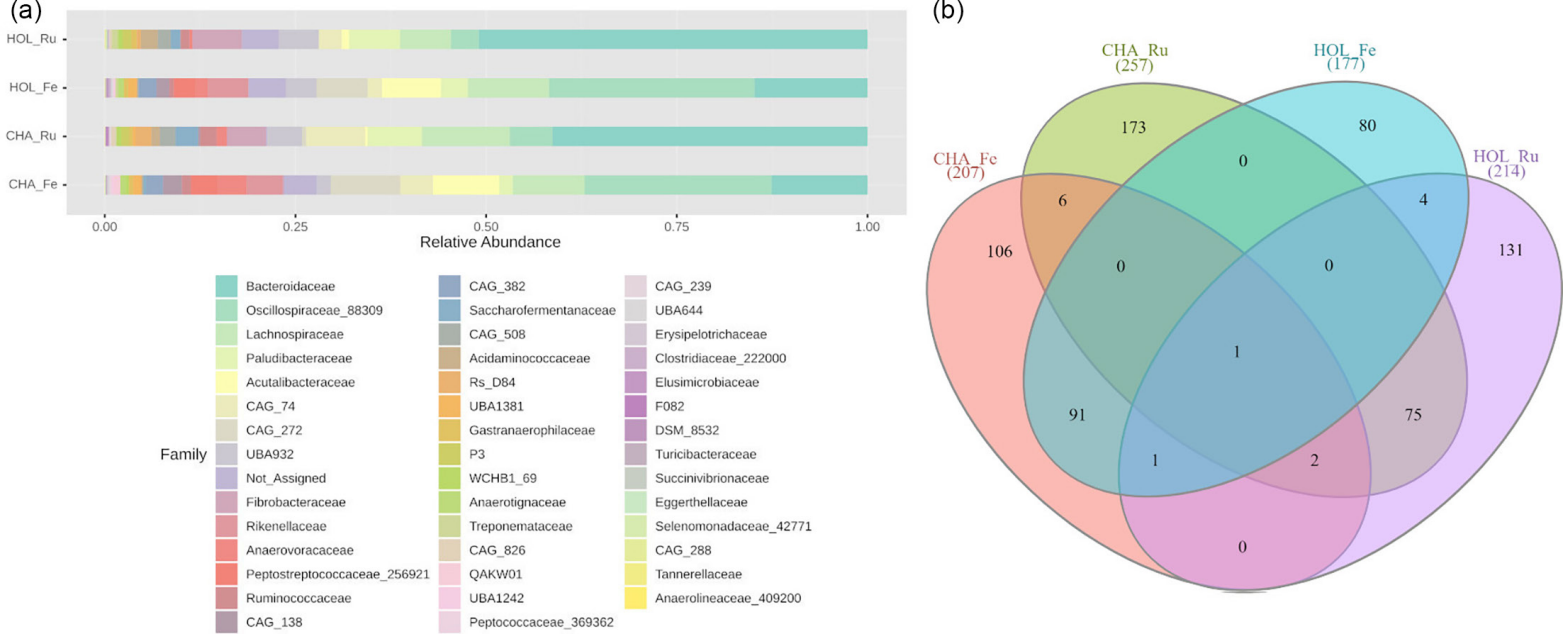
Mean relative frequency of taxa at family level (**a**) and number of core ASVs (**b**) across the four groups, namely CHA_Ru, CHA_Fe, HOL_Ru and HOL_Fe.

At the phylum level, on average, 70% of ASVs from faecal samples were classified as *Bacillota* (formerly *Firmicutes*), while 28% were *Bacteroidota* (formerly *Bacteroidetes*) (Fig. 1). Conversely, in rumen samples, the proportion was reversed, with 59% of ASVs classified as *Bacteroidota* (such as *Bacteroidaceae* and *Paludibacteraceae* families) and 28% as *Bacillota* (such as *Lachnospiraceae* and *CAG-74* families). Other phyla were *Fibrobacterota* (4%), *Pseudomonadota* (3%) and *Cyanobacteriota* (2%) (Fig. 1a, Table S1; check Fig. S1 for per-sample results). Regarding the core microbiome, there were more core ASVs in the rumen than in faeces, and higher numbers were detected in CHA than in HOL. We identified 207, 257, 177 and 214 ASVs that were present in at least 50% of the samples in CHA_Fe, CHA_Ru, HOL_Fe and HOL_Ru, respectively (Fig. 1b). Despite differences in diet, location and breed, up to 35% of the rumen ASVs are shared between groups, and this number increases to 44% in terms of common faecal ASVs (Table S2). A limited cross-site overlap of core microbes exists between rumen and faeces within groups, which is expected and highlights extreme niche specialization. Only one ASV was present in all groups and corresponds to *Mogibacterium*, a Gram-positive bacterium within the *Bacillota* phylum.

**Fig. 2.**
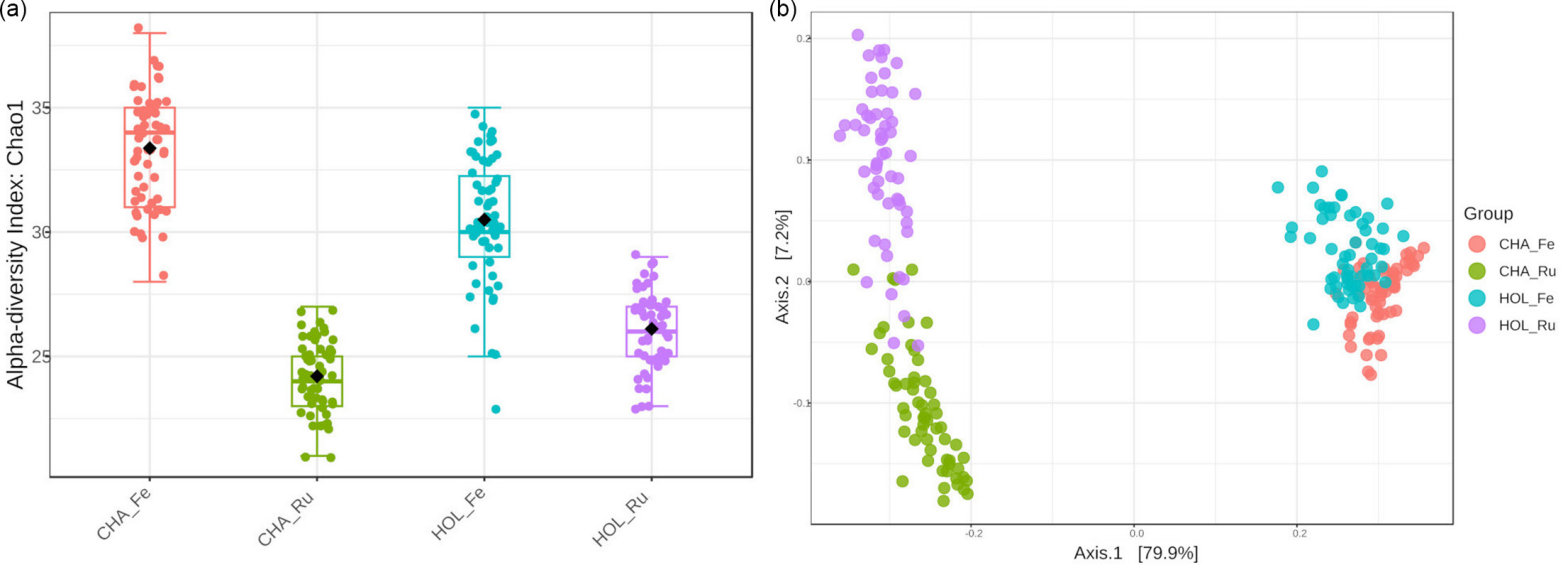
Alpha (Chao1 index) (**a**) and beta (Bray–Curtis) (**b**) bacterial diversity at the family level, with each point representing an individual sample from CHA_Ru, CHA_Fe, HOL_Ru and HOL_Fe groups.

At the family level, alpha-diversity (Chao1) was higher for faeces in both production systems compared to rumen (Fig. 2a). While no difference was found between rumen datasets (FDR=0.31), higher alpha-diversity was observed in Charolais faeces compared to Holstein’s (FDR=7.78E−10). Within-breed differences between rumen and faeces were also significant (FDR<1E−15). A PCoA based on beta-diversity (Bray–Curtis) (Fig. 2b) shows a clear distinction between sampling sites, accounting for 79.9% of the variation. At the same time, differences between production systems captured by PC2 were less evident, particularly for faeces, accounting for 7.2% of the variation.

### Network architecture and ASV–phenotype associations

**Fig. 3.**
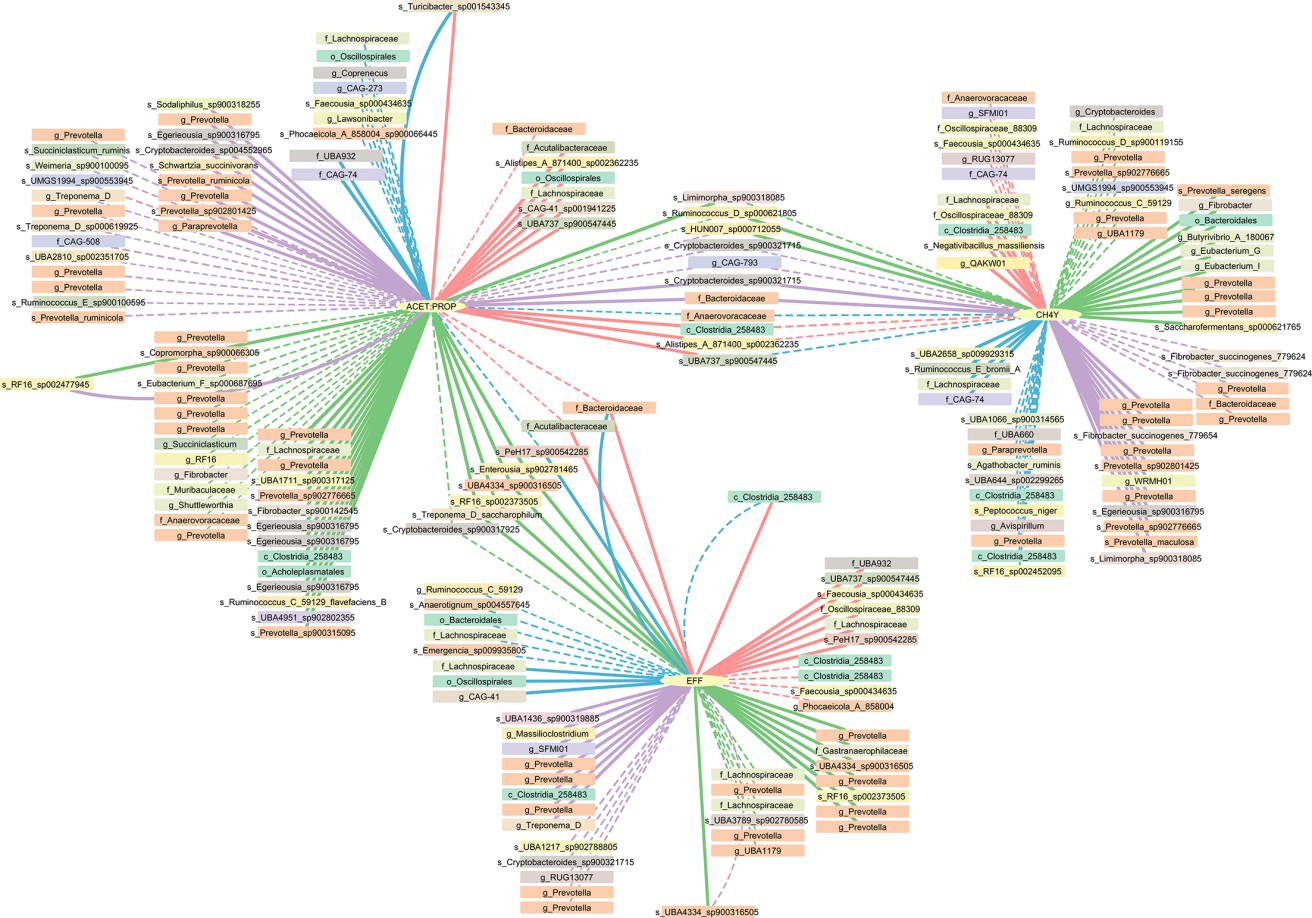
Combined network relating phenotypes to ASVs. Rectangular nodes represent unique ASVs, named by their lowest assigned taxonomic classification and coloured according to family. Solid and dashed edges represent positive and negative correlations, respectively. Edge colours represent the network of origin, namely CHA_Ru (green), CHA_Fe (pink), HOL_Ru (purple) and HOL_Fe (blue). File S7 provides these results in text format.

After filtering out ASVs present in fewer than 20 samples, we constructed 4 co-association networks using the three phenotypes (EFF, CH4Y and ACET:PROP) and the resulting ASVs for CHA_Fe (*n*=320), CHA_Ru (*n*=387), HOL_Fe (*n*=277) and HOL_Ru (*n*=341). Although Partial Correlation and Information Theory analysis explore the relationship among all features in the network, for the objective of this work, we focused only on the direct connections between an ASV and a phenotype. Fig. 3 summarizes the results for all four networks, and more details can be found in Tables S3–S6. Correlation strengths range from ~0.2 to ~0.4, indicating moderate but significant associations. Not surprisingly, given the nature of the phenotypes, a larger number of connections was found in the rumen networks, with 77 ASVs in Charolais and 62 ASVs in Holstein. Connections in faeces networks totalled 39 in Charolais and 40 in Holstein. Overall, the phenotype with the higher number of connections was ACET:PROP (87), followed by CH4Y (72) and EFF (57). Both positive and negative correlations were identified for all phenotypes and networks (Fig. 3, solid and dashed lines, respectively).

### Taxonomic enrichment in phenotype-associated ASVs

Analysis of ASVs directly connected to phenotypes revealed distinct taxonomic enrichment patterns across networks (Table 2, Table S7). Rumen networks showed clear family-level enrichments, while faecal networks demonstrated limited taxonomic clustering of phenotype-associated ASVs. For instance, *Bacteroidaceae* emerged as the most consistently enriched family, appearing in associations across all phenotypes in both rumen networks, regardless of production system. However, examination of correlation directions revealed that *Bacteroidaceae* ASVs showed mixed associations with production traits, with both positive and negative correlations observed within this family across all datasets where enriched. Similarly, *Ruminococcaceae* and *Lachnospiraceae* were prominently enriched in CHA_Ru networks across multiple phenotypes and demonstrated mixed correlation patterns, which suggests functional diversity rather than uniform effects. *Fibrobacteraceae* showed specific enrichment for ACET:PROP in CHA_Ru and CH4Y in HOL_Ru, displaying mixed correlations in both cases. *CAG-508* was notable as one of the few enriched families showing consistent directional associations, displaying exclusively negative correlations in HOL_Ru networks. In contrast, faecal networks showed minimal taxonomic enrichment, with only *Oscillospiraceae_88309* (enriched for CH4Y in CHA_Fe) and *Acutalibacteraceae* (enriched for ACET:PROP in CHA_Fe) detected, and no significant enrichments in HOL_Fe for any phenotype.

**Table 2.** Enrichment at the family level of ASVs associated with the phenotypes EFF, CH4Y and ACET:PROP within each dataset, namely CHA_Ru, CHA_Fe, HOL_Ru and HOL_Fe

Dataset	Phenotype	Enriched taxa (score*)
CHA_Ru	EFF	*Paludibacteraceae* (2.98); *Bacteroidaceae* (1.50)
	CH4Y	*Ruminococcaceae* (2.94); *Lachnospiraceae* (2.76); *Bacteroidaceae* (0.86)
	ACET:PROP	*Ruminococcaceae* (2.82); *Fibrobacteraceae* (2.67); *Lachnospiraceae* (2.22); *Bacteroidaceae* (0.17)
CHA_Fe	EFF	–
	CH4Y	*Oscillospiraceae_88309* (1.86)
	ACET:PROP	*Acutalibacteraceae* (3.00)
HOL_Ru	EFF	*Bacteroidaceae* (2.12)
	CH4Y	*Fibrobacteraceae* (2.76); *UBA932* (2.54); *Bacteroidaceae* (1.10)
	ACET:PROP	*CAG-508* (2.69); *UBA932* (2.15); *Bacteroidaceae* (0.71)
HOL_Fe	EFF	–
	CH4Y	–
	ACET:PROP	–

* Enrichment scores indicate statistical over-representation of taxonomic families relative to background expectations, with higher scores representing greater enrichment [hypergeometric *P*(*x*≥*X*) values].

### Predictive performance of selected vs. random ASVs

Network-selected ASVs outperformed randomly selected ASVs when using the same number of variables (Table 3, Table S8). The full models, which include all ASVs directly connected to phenotypes in each network, explained 47–88% of the phenotypic variance and consistently outperformed random selection of an equivalent number of ASVs. Performance differences between full models and random selection ranged from 0.18 (ACET:PROP in CHA_Ru) to 0.44 (EFF in HOL_Ru), with an average improvement of 0.31 *R*² units, demonstrating that network-identified ASVs capture genuine biological signal rather than spurious correlations. As anticipated, CV-*R*² was lower than model-fit *R*² and displayed fold-to-fold variability due to the ~11-animal validation folds (see Table S8 for per-fold CV-*R*²). Nevertheless, several settings showed non-trivial CV signal (e.g. CHA_Fe EFF mean CV-*R*²=0.54; fold values: 0.47, 0.42, 0.33, 0.70, 0.76), whereas others were modest (e.g. CHA_Ru ACET:PROP mean CV-*R*²=0.36; HOL_Fe ACET:PROP mean CV-*R*²=0.11).

**Table 3.** Multiple regression results using all ASVs directly connected to phenotypes in the network (full model), using ASVs selected randomly (Random Selection), using selected ASVs based on the RSQUARE method (Selection) and a 5-fold cross-validation (CV) based on the selected ASVs

Dataset	Trait	Full model			Random selection			Selection			Cross-validation	
		N ASVs	*R* ^2^		N ASVs	*R* ^2*^		N ASVs	*R* ^2^		N ASVs	CV-*R*^2*^
CHA_Ru	EFF	19	0.61		19	0.36		8	0.58		8	0.30
	CH4Y	22	0.62		22	0.39		11	0.59		11	0.17
	ACET:PROP	36	0.88		36	0.71		17	0.84		17	0.36
CHA_Fe	EFF	13	0.63		13	0.19		8	0.59		8	0.54
	CH4Y	13	0.59		13	0.24		7	0.56		7	0.46
	ACET:PROP	13	0.57		13	0.25		6	0.54		6	0.24
HOL_Ru	EFF	14	0.70		14	0.26		8	0.66		8	0.27
	CH4Y	20	0.63		20	0.36		7	0.60		7	0.20
	ACET:PROP	28	0.81		28	0.54		15	0.76		15	0.23
HOL_Fe	EFF	11	0.47		11	0.20		7	0.45		7	0.24
	CH4Y	17	0.69		17	0.33		9	0.65		9	0.11
	ACET:PROP	12	0.61		12	0.22		8	0.58		8	0.11

*Random selection: mean *R*² across ten independent random draws. CV: mean CV-*R*² across the five folds of a single 5-fold split. See Table S8 for fold-wise CV-*R*² and per-draw random values.

Finally, the RSQUARE method enabled substantial model simplification without meaningful loss of predictive accuracy. We were able to select using 33–65% fewer ASVs (6–17 vs. 11–36 in full models) while retaining 93–97% of full model performance across all phenotype–dataset combinations. For example, for ACET:PROP in CHA_Ru, we achieved 95% of full model performance (*R*²=0.84 vs. 0.88) using less than half the ASVs (17 vs. 36), indicating potential significant functional redundancy within phenotype-associated microbial communities.

## Discussion

This study sought to identify gastrointestinal microbiota from the rumen and faecal ecosystems associated with feed efficiency and methane emissions in Holstein and Charolais cattle. By combining microbial co-abundance networks with multiple regression, we identified sets of ASVs that supported the prediction of ACET:PROP (as a proxy for CH4), EFF and CH4Y. Across all datasets, network-selected ASVs provided substantially better predictive performance than non-targeted selections, indicating that the identified associations reflect meaningful biological signal rather than spurious correlations. The RSQUARE procedure further enabled considerable model simplification, retaining 93–97% of the model-fit *R*² while using 33–65% fewer ASVs, consistent with functional redundancy within phenotype-associated microbial communities. Cross-validation, however, showed variable performance across folds due to the small validation subsets (~11 animals), underscoring the exploratory nature of these predictions and the need for evaluation in larger and more diverse populations.

We observed a higher number of associations in the ruminal rather than the faecal ecosystem for both CHA and HOL. This pattern was most pronounced for the ACET:PROP ratio, which showed the highest predictive accuracy across all datasets (*R*²=0.54–0.84), reflecting its direct microbial origin. Notably, in the Holstein dataset, the stronger negative association with the ACET:PROP ratio (*r*=−0.42) was of ASV (d25771f7b9618951975a3732f801efcc) from the genus *UBA2810*, family *Succinivibrionaceae*. Since a higher ACET:PROP ratio is often associated with higher CH4 production in the rumen, this observation is consistent with the well-known negative associations between members of the *Succinivibrionaceae* family and CH4 emissions [11,21,24]. Moreover, it is noteworthy that *UBA2810* has recently been proposed as a keystone genus driving ruminal microbial guilds (RuminoSignatures) that are negatively associated with CH4 and carbon dioxide (CO₂) emissions in dairy cattle [25]. The higher performance of randomly selected ASVs observed for ACET:PROP in rumen samples (*R*²=0.54–0.71) reflects the strong overall microbial signal for VFA production, where even randomly selected ASVs capture some fermentation-related variation. This result underscores the central role of ruminal microbial communities in VFA metabolism and highlights the superior performance of network-informed selection.

Most of the observed associations among selected ASVs were breed-specific, with only a few exceptions, including a negative correlation between the ACET:PROP ratio and ruminal ASVs belonging to the *Prevotella* and *Ruminococcus* genera, as well as positive links with members of the *Prevotella* and *Egerieousia* (*Bacteroidales* order) genera. Concerning CH4Y, the ASVs positively associated included, in addition to *Prevotella* and *Egerieousia*, members of the *Fibrobacter* genus. Conversely, negative associations with CH4Y were evident for ASVs belonging to *Prevotella* and *Cryptobacteroides*. A similar pattern emerged for EFF, characterized by microbial signatures that showed a negative association with members of *Prevotella* and *Cryptobacteroides*. Notably, EFF showed the most variable predictive performance across datasets (*R*²=0.45–0.66), suggesting complex polygenic and polymicrobial determination with strong environmental (e.g. diet) influences beyond direct microbial effects [26]. Our results also revealed stronger associations within the rumen compared to faeces, particularly for VFAs and CH4, which are directly influenced by microbial activity. However, no direct ASV connections were detected between CH4 emissions and feed efficiency (Fig. 3), suggesting that their relationship may be mediated by other metabolic or host factors rather than specific microbial taxa.

Analysis of enriched taxonomic families revealed that most showed mixed correlation patterns within the same taxonomic groups. *Bacteroidaceae*, *Ruminococcaceae* and *Lachnospiraceae* all demonstrated both positive and negative correlations with production traits, indicating significant functional diversity within these families rather than uniform effects. For example, we observed that *Prevotella* ASVs can be positively and negatively correlated with both CH4Y and ACET:PROP ratio, in agreement with previous studies [21,22, 27]. This can be explained by the known functional diversity within the genus [28] and supports ASV-level analysis for predictive applications rather than grouping by taxonomy when lower levels of classification cannot be achieved, which can be a limitation of 16S rRNA data.

Selected faecal ASVs were even more breed and trait-specific, and taxonomic classification at the genus level was scarcer. For example, associations with EFF in Charolais included positive correlations with families *Oscillospiraceae* (genus *Faecousia*), *Acutalibacteraceae* and *UBA932* and negative correlations with *Bacteroidaceae* (genus *Phocaeicola*), *Oscillospiraceae* (genus *Faecousia*) and *CAG-138* (genus *PeH17*), while for Holstein, positive correlations include *UBA1381* (genus *CAG-41*) and *Lachnospiraceae*, and negative correlations include *Anaerovoracaceae* (genus *Emergencia*), *Lachnospiraceae*, *Ruminococcaceae* (genus *Ruminococcus*) and *Anaerotignaceae* (genus *Anaerotignum*). Although in both production systems members of the *Lachnospiraceae* family were associated with CH4Y, in Charolais the association is positive, while in Holstein it is negative.

In Holstein, EFF exhibited stronger associations with the rumen microbiota compared to faeces, potentially reflecting the rumen’s central role in nutrient partitioning for milk production. In contrast, Charolais showed similar but weaker rumen and faecal microbial links to EFF. This divergence may explain the inconsistent microbiota-feed efficiency and CH4 correlations reported in the literature [29,30] and highlights how gastrointestinal microbial ecosystems are shaped by production systems, diet and breed-specific factors that are key distinctions between the datasets used in this study.

Consistent with previous findings [24], our results confirm the presence of a core microbiota in ruminants and further demonstrate that this core extends to the faecal microbiota. Most of the ASVs associated with our three traits were considered core microbiome, but this was specific to each dataset. For instance, cellulolytic bacteria *Ruminococcus*, previously shown to be part of the ruminal heritable core microbiome [24], were here considered predictive of CH4Y in Charolais and ACET:PROP in both Charolais and Holstein. Similarly, *Fibrobacter* was predictive of CH4Y and ACET:PROP in Charolais, and although associated with CH4Y in Holstein, it was not included among the final selected taxa. Just a handful of ASVs were shared between the rumen and faecal microbiota within each breed, and only one ASV (38d8434af42eefe708173cb956e2b184) classified as *Mogibacterium* was observed as core across all four datasets. *Mogibacterium* is a Gram-positive bacterial genus that has been previously shown to be significantly decreased in animals with low methane emissions and is negatively associated with the percentage of propionate [31]. However, in our study, this ASV shows no predictive potential to any of the phenotypes and datasets.

Regarding the practical applications of this work, the substantial model simplification achieved (using ~50% fewer ASVs while retaining >90% performance) suggests potential for developing rapid, cost-effective screening panels for breeding programmes. However, the production system-specific nature of associations indicates that universal screening tools may be limited, requiring context-specific calibration for different breeds and management systems. In addition, given that cross-validation showed modest and sometimes unstable predictive performance in the small held-out folds, validation of these results in larger or independent populations is required. Future research should focus on longitudinal sampling, exploring alternative approaches to identify microbial guilds [32] and integrating multi-omics data, such as metaproteomics or metabolomics, for functional validation. The potential for targeted interventions focusing on selected keystone taxa, rather than whole-community manipulations, represents a promising avenue for precision livestock management.

## Conclusion

This study demonstrates that cattle gastrointestinal microbiomes contain promising predictive microbial signatures for production traits, with rumen communities showing superior predictive potential compared to faecal communities. Using network-based approaches, we successfully identified keystone microbial taxa that achieve high predictive accuracy with fewer variables than conventional methods, providing evidence for significant functional redundancy within phenotype-associated microbial communities. However, while our network-guided predictors show biological coherence and promising model fit performance, their generalization will require validation on independent or larger datasets. In addition, contrary to our initial hypothesis that we would identify common predictive micro-organisms across production systems by analysing two datasets under the same pipeline, we found that microbial–phenotype associations were predominantly production system-specific, with limited overlap between Holstein dairy and Charolais beef cattle. This finding highlights the importance of considering breed, diet and management context when developing microbiome-based breeding tools.

## Abbreviations

ACET:PROP: acetate/propionate ratio
ASVs: amplicon sequence variants
CHA: Charolais heifers
CHA_Fe: Charolais faeces
CHA_Ru: Charolais rumen
CH4Y: methane yield
CLR: centred log-ratio
CSS: cumulative sum scaling
CV: cross-validation
DMI: dry matter intake
EFF: feed efficiency
HOL: Holstein cows
HOL_Fe: Holstein faeces
HOL_Ru: Holstein rumen
OTUs: operational taxonomic units
RV: vector correlation coefficient
VFAs: volatile fatty acids
